# Generation and evaluation of polyclonal antibodies specific for ToxA from *Vibrio parahaemolyticus *causing acute hepatopancreatic necrosis disease (AHPND) in shrimp

**DOI:** 10.22099/mbrc.2020.38774.1561

**Published:** 2021-03

**Authors:** Khai-Hoan Nguyen-Phuoc, Ngoc-Diem Duong, Thach Van Phan, Kim-Yen Thi Do, Nguyet-Thu Thi Nguyen, Thuoc Linh Tran, Hieu Tran-Van

**Affiliations:** 1 Department of Molecular and Environmental Biotechnology, Faculty of Biology and Biotechnology, University of Science, Ho Chi Minh, Vietnam; 2 Vietnam National University, Ho Chi Minh, Vietnam; 3 Pasteur Institute in Ho Chi Minh City, Vietnam; + First authorship shared

**Keywords:** AHPND, *Vibrio parahaemolyticus*, Polyclonal antibodies, Recombinant toxin, ToxA; Immuno-interactions

## Abstract

Acute Hepatopancreatic Necrosis Disease (AHPND) is a newly emerging shrimp disease with mortality up to 100 percent caused by *Vibrio parahaemolyticus* which carries a plasmid encoding for two toxins, ToxA and ToxB. In 2013, the Global Aquaculture Alliance (GAA) estimated shrimp farming decline in Asia accounted for 1-billion US dollar lost. Currently, diagnosis using PCR method does not meet the demand of *in situ* detection, which is based on antigen-antibody interaction, has not been developed yet. In this present study, we proceeded to create the toxin and its antibody for lateral flow development. First, recombinant toxin ToxA was generated by gene manipulation. After that, purified ToxA was used to immunize rabbits. Finally, antisera from rabbits and protein-A purified antibodies were evaluated for titer, specificity, and detection threshold. Results showed that recombinant ToxA was overexpressed in soluble fraction at 37^o^C with 1mM IPTG. Purification by affinity chromatography was able to isolate recombinant ToxA with the purity up to 94.49%. In ELISA experiment, the immunized antisera reached a titer of up to 1/5,210,000 with 1µg/ml of antigen, and detection threshold was 100ng recombinant toxin. After purification, the detection threshold of purified polyclonal antibodies was 25ng toxin per dot. These results laid a groundwork for the development of AHPND detection kit based on antigen - antibody interactions.

## INTRODUCTION

In 2018, shrimp production was estimated at more than 4 million tons (FAO) [1], with an export value of more than 19 billion US dollar, hence shrimp farming has a great potential. However, shrimp farming faces many diseases [2, 3], such as white spot disease [4], yellow head disease [5, 6], and a newly emerging disease named acute hepatopancreatic necrosis (AHPND). AHPND was first discovered in China in 2009 [7], spread to Vietnam in 2010, and continued to spread to Malaysia and Thailand in 2011 and 2012 [8, 9], Mexico in 2013 [10]. After that, AHPND also spread to the Philippines in 2015 and South America in 2016 [11], and first reported in the United State of America in 2017 [12] . The disease spreads quickly and causes death with mortality rate up to 100% within 30-35 days of pond-stocking [13-15]. Therefore, AHPND has caused huge damage to shrimp farming, from 2009 to 2016 shrimp production losses due to AHPND has been reported is estimated to be about 23 billion US dollars [16] and AHPND caused a sharp decline in shrimp production. In Vietnam, AHPND is the leading cause of damage to shrimp farming. The disease appeared in many provinces, affected about 39,000 ha of shrimp farming, causing damage of about 100 million US dollars [13]. The way to effectively prevent AHPND spreading is to develop a fast and early detection kit. 

In 2013, the cause of AHPND was confirmed as *Vibrio parahaemolyticus* [17]. Then, studies in 2014 and 2015 indicated that only strains carrying plasmid coding for two toxins ToxA (13kDa) and ToxB (50kDa) could cause disease [18-20]. Base on this discovery, many PCR kits were developed to detect AHPND. Primers such as AP1, AP2 [21], AP3 [22] for one step PCR or AP4 for nested PCR [23], and multiplex PCR using primers VpPirA-284 and VpPirB-392 [24] opened the door for AHPND detection because of their high sensitivity and accuracy. However, PCR method takes time to get a result. It also requires equipment and laboratory for processing PCR test, thus farmers have to send their samples to diagnosis centers. This in turn wastes critical time for preventing the spread of AHPND. Therefore, a fast, simple *in situ* test kit is a meet need for the farmers in this situation which lateral flow assay (LFA) kit comes to the place [25]. The assay was successfully developed for diagnosis of white spot syndrome virus [26] and yellow head virus [27]. LFA bases on specific interactions between antigen and antibody, and in this case ToxA and ToxB were the antigens of focus in detection AHPND by LFA. In 2017, monoclonal antibodies against two toxins were generated. Threshold of these antibodies was 3ng for recombinant ToxA, and 0.78ng for ToxB, respectively [28]. Moreover, the cost of monoclonal antibody is quite high, and the process of production is quite complicated, which leads to increase the total cost for LFA mass production. Thus, polyclonal antibody is an alternative in this condition, because of low cost in production and easier in generation. 

Recent study predicts that the function of ToxA is to bind to shrimp cell(s) and initiate pathogenesis process [29, 30]. This makes ToxA is the first target for AHPND detection. Therefore, in this present study, polyclonal antibodies specific for ToxA toxin of *Vibrio parahaemolyticus* causing AHPND in shrimp were generated and evaluated. These antibodies will be the source for lateral flow assay kit development based on immuno-interactions.

## MATERIALS AND METHODS

**Strains of bacteria, and plasmids:** *Vibrio parahaemolyticus* strains causing AHPND was provided by the Research Sub-Institute for Aquaculture in Nam Song Hau, Vietnam. *Vibrio parahaemolyticus* AHPND strain XN89 (VP_AHPND_), *Vibrio parahaemolyticus *non-AHPND strain XN8 (VP_non-AHPND_) served as positive and negative controls, respectively was kindly provided by Dr. S. Senapin, National Center for Genetic Engineering and Biotechnology (BIOTEC), Thailand.* Vibrio cholerae, Vibrio vulnificus, Vibrio alginolyticus*, and White Spot Syndrome virus was kindly provided by Dr.Tuan V. Vo from University of Agriculture and Forestry, Ho Chi Minh City, Vietnam. *Escherichia coli* DH5α and *Escherichia coli* BL21(DE3) were used as strains to replicate recombinant vectors and to express recombinant protein, respectively; pET22b plasmid was used for cloning as well as target gene expression driven by T7 promoter through IPTG inducer.

**Generation of** ***E. coli*** **BL21(DE3) strain carrying recombinant vector pET22b-toxA: **The gene coding for ToxA toxin (*toxA*) was obtained from *V. parahaemolyticus* AHPND strain XN89 by PCR method with specific primers, toxA-F and toxA-R, negative control was a PCR without DNA template from *V. parahaemolyticus*. Next, *toxA* gene and plasmid pET22b were digested with two restriction enzymes, *Nde*I and *Xho*I and ligating using T4 ligase. The ligated product was transformed into *E.coli* DH5 and cultured on Luria broth (LB) agar containing 100µgmL-1 ampicillin and incubated overnight at 37^o^C. After incubated, colonies on LB agar were screened by PCR with toxA-F and T7ter primers (pairing at the T7 terminator region on the plasmid pET22b), recombinant vector was collected from a positive colony and sequenced. After that, recombinant vector pET22b-*toxA *was transformed into *E. coli* BL21(DE3) and cultured on LB agar medium with 100µgmL-1 ampicillin and screened with the same procedure as *E.coli* DH5. All results of PCR method were analyzed by electrophoresis on agarose 1.5% with 1kb ladder maker (Bioline, UK).

**Expression of recombinant ToxA: **Strain of *E.coli *BL21(DE3)/pET22b-*toxA* was cultured in 5mL LB medium containing ampicillin (100µgmL-1), at 37°C, for 16 hours. Then, sub-cultured bacteria at 1:20 (v/v) dilution continued at 37°C until OD_600_ reached 0.6 to 0.8. IPTG was added into the culture medium with a final concentration of 1 mM and cultured at 37°C, for 4 hours. Next, 1.5mL bacterial broth was pelleted, mixed with 300µL solution A (20 mM phosphate, 0.5M NaCl, pH 7,4), sonicated and subsequently centrifuged to collect total protein, soluble protein, and insoluble protein fractions. Expression of the recombinant protein was confirmed by SDS-PAGE with Coomassie Blue staining and Western blot probed with 6xHis antibody. Control samples were total fraction of *E. coli* BL21(DE3) with IPTG induction, *E. coli* BL21 (DE3)/pET22b with IPTG induction and *E.* *coli* BL21(DE3)/pET22b-*toxA* non-IPTG induction.

**Purification of recombinant ToxA by affinity chromatography: **The expression of *E.coli *BL21 (DE3)/pET22b-*toxA* strain was made with the scale of 100mL LB medium. Soluble protein fraction was purified by affinity chromatography with Histrap^TM^ column HP (GE Healthcare). The column was equilibrated with solution A, then was loaded with total protein fraction. Next, the column was re-equilibrated with solution A and washed with solution B (20 mM phosphate 0.5M NaCl, 90 mM imidazole, pH 7.4). Finally, the target protein was eluted with solution C (20 mM phosphate, 0.5M NaCl, 108 mM imidazole, pH 7.4). After purification, protein ToxA was concentrated and changed to PBS buffer using 10 kDa MWCO concentrator. Result of purification ToxA was verified by SDS-PAGE with silver staining, and analyzed by Gel Analyzer software.

**Production of polyclonal antibody against ToxA: **All rabbits were maintained in the experimental animal facility, and experiments were performed in accordance with the guidelines provided by the Animal Care and Use Committee of Pasteur Institute in Ho Chi Minh City. Briefly, one hundred μg of recombinant ToxA in 1mL of NaCl 0.9% and 1mL of Complete Freund's Adjuvant (CFA) were emulsified for primary immunization. Each 2mL emulsion was immunized into 3 male rabbits. Before immunization, rabbit serums were collected as negative controls for antibody titer evaluation. Every 4 weeks, the rabbits were given immunization with Incomplete Freund's Adjuvant (IFA) and the amount of ToxA was reduced to 75µg for the first repetition and 50µg for the next three repetitions. Rabbit antiserum were obtained two weeks after each injection.

**Evaluating antiserum titer: **Antiserum titer was evaluated by ELISA[31]. Recombinant ToxA was mixed with 0.1M carbonate buffer, pH 9.6 with final concentration of 1µgmL-1 and incubated 100µL each well at room temperature overnight. After that, the ELISA plate was washed four times with PBS-T (PBS with 0.05% Tween). Next, wells were blocked with 5% skim milk in PBS, incubated at 37°C for 1 hour and washed 4 times with PBS-T. Then, diluted concentrations from 1/5,000 to 1/5,210,000 of antiserum in PBS with 1% skim milk were added and incubated for 1 hour at 37°C. Diluted pre-bleed at 1/5,000 was used at negative control. After washing four times with PBST, HRP conjugated goat anti-rabbit IgG secondary antibody was added and incubated at 37°C for 1 hour before washing step with PBS-T. Finally, TMB substrate was added to all wells, incubated for 5 mins before adding 100µL stop solution (H_2_SO_4_ 2N). Results were read by ELISA (Multikan Ascent) plate reader at 450nm.

**Specificity evaluation of antiserum: **Proteins from strains VP_AHPND_, VP_non-AHPND_, total protein *E.coli* BL21(DE3)/pET22b-*toxA* and purified recombinant ToxA protein were used for specificity evaluation of antiserum by Western blot. Proteins were separated by SDS-PAGE and transferred to nitrocellulose membranes. After blocking with 3% skim milk, antiserum was diluted to 1/10,000 and incubated for 1 hour. Then HRP conjugated goat anti-rabbit IgG was added and incubated for 1 hour. Finally, TMB substrate was added and incubated for 15 minutes. Results on membrane was digitalized documented.

In addition, the specificity of antiserum was evaluated by Dot blot method with strains *V. cholerae, V. vulnificus, V. alginolyticus*, and White Spot Syndrome virus. Cell lysis of bacteria were fixed onto nitrocellulose membrane and next steps were conducted as the same as Western blot method. The color dots on membrane were digitalized documented.

**Purification of polyclonal antibodies and sensitivity evaluation: **Polyclonal antibodies were collected from antisera by combining two methods, precipitation with 50% ammonium sulphate and chromatography with protein A column. The purity was analyzed by SDS- PAGE and silver staining. Antiserum and polyclonal antibodies were evaluated for sensitivity by Dot blot method with three difference samples including recombinant ToxA protein, protein from VP_AHPND_ and VP_non-AHPND_. The samples were diluted in two-fold dilution manner, starting from 800ng, and fixed onto nitrocellulose membrane. Next steps were performed as the same as Western blot method. The color dots on membrane were digitalized documented.

## RESULTS 

**Figure 1:**
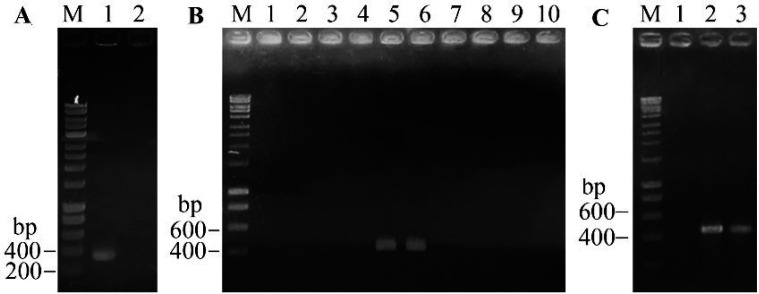
Results of electrophoresis on agarose gel. (A) Isolation of gene *toxA. *M, ladder. Lane 1, gene toxA. Lane 2, negative control. (B) Screening colonies from pET22b-*toxA* candidates. M, ladder. 1, negative control. 2-10, candidate colonies. (C) Screening BL21(DE3)/pET22b-*toxA* strains. M, ladder. 1, negative control. 2-3 candidate colonies

Gene encoding for toxA was obtained from the AHPND using PCR method and checked by electrophoresis on 1.5% agarose gel. The results of electrophoresis showed a single band (lane 1, Fig. 1A), and there was no band in a negative control on gel. After ligation and transformation, colonies were screened by PCR with toxA-F and T7ter primers. As expected, the toxA gene would be inserted into a region between T7 promoter and T7 terminator of plasmid pET22b, thus an amplification sequence length was 446bp. The electrophoresis results showed positive colonies with predicted band (lanes 5, 6, Fig. 1B). In addition, the negative control did not show any band, indicating that no contamination occurred during PCR (lane 1, Fig. 1B).

The result was confirmed again by sequencing plasmids obtained from positive colonies. Sequencing result showed that a typical recombinant clone obtained was 100% homologous to 3HP pathogenic strain published in the GenBank [18, 32] (data not shown).

**Figure 2:**
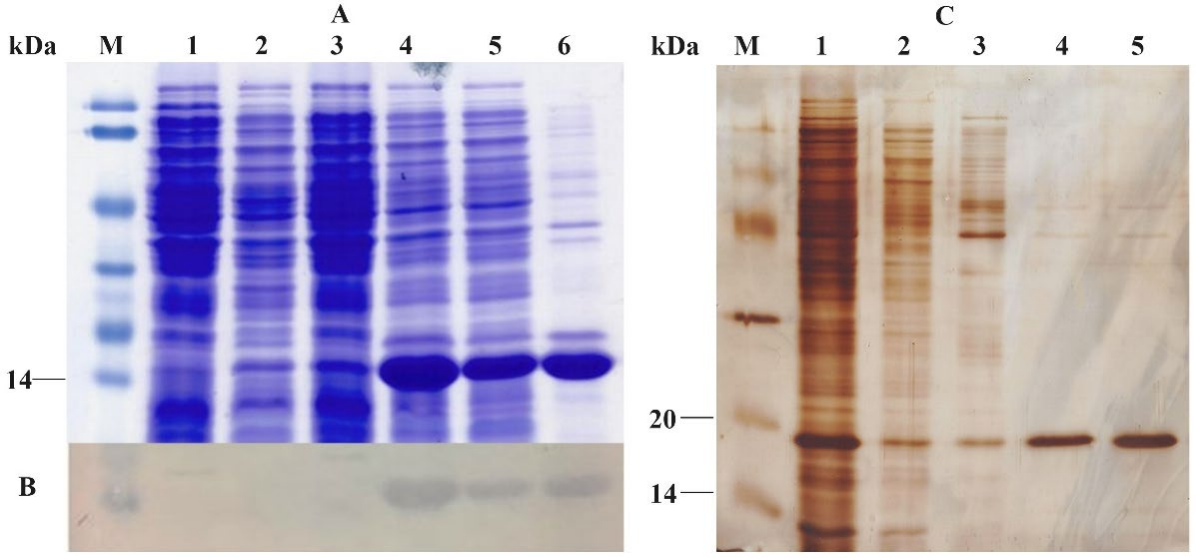
Results of SDS-PAGE (A), Western blot (B) of expression and purification (C) ToxA. In (A) and (B) M, protein ladder. 1, *E. coli* BL21(DE3) (+1mM IPTG). 2, *E. coli* BL21(DE3)/pET22b (+1mM IPTG). 3, *E. coli* BL21(DE3)/pET22b-toxA (-IPTG); 4-6. *E.coli* BL21(DE3)/pET22b (+IPTG) total, soluble, insoluble proteins. (C) M, protein ladder. 1, total protein. 2, flow-through fraction. 3 washing fraction. 4-5, elution fractions

In *E. coli* BL21(DE3)/pET22b-toxA strain, ToxA protein was overexpressed as a recombinant protein about 14kDa (lane 5, Fig. 2A), a predicted size of ToxA, in soluble fraction. Also, there was no band of target protein in control samples. Next, the expression of ToxA was confirmed indirectly using Western blot probed with anti-6xHis antibody. Western blot result showed that the overexpressed protein in SDS-PAGE gel was ToxA protein with 6xHis fusion (Fig. 2B).

After that, ToxA protein was purified by Ni^2+^ chromatographic column. Then, purified fractions were analyzed using SDS-PAGE and Gel Analyzer software. As shown in Figure 2C, flow through and washing fractions (Fig. 2C, lanes 2-3) appeared many protein bands, including partial ToxA protein. Evaluation by Gel Analyzer showed that ToxA protein in elution fraction had a purity of more than 94%, and recovery efficiency of purification process was 40.29%.

**Table 1:** Quantity and recovery efficiency of purification protein ToxA

	**Volume (ml)**	**Concentration** **(µg/ml)**	**Percent of protein (%)**	**Quantity of protein (µg)**	**Recovery efficiency (%)**
Total protein Purified protein	20	1808	21.01	7235.62	
	1.5	2088	94.49	2959.43	40.29

**Figure 3:**
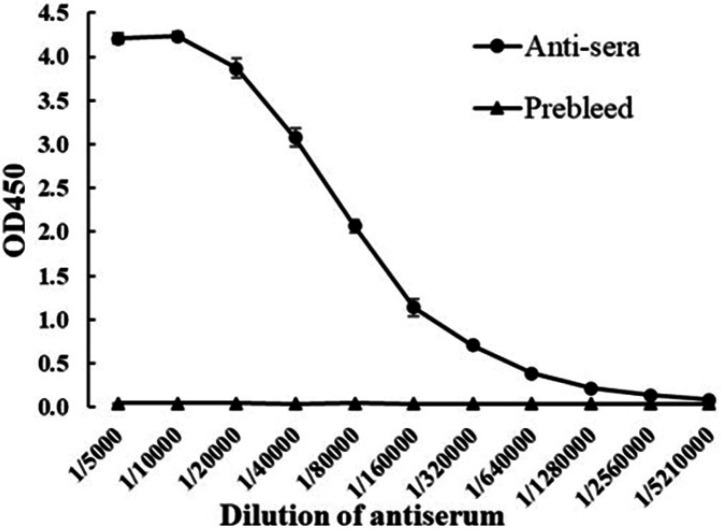
ELISA results of evaluation antiserum titer

Antibody titer evaluation was performed by indirect ELISA with 1µgmL-1 antigen and antiserum diluted from 1/5,000 to 1/5,210,000. Signals were measured at 450nm and graphed in Figure 3. The antiserum titer was defined as the highest dilution that still gives a positive value. The result showed that antiserum gave a high signal from 1/5,000 and 1/10,000 dilutions. After that, the signal began gradually declined at next dilutions and reached the cut-off value (mean ± 3SD) [33] at 1/5,210,000 dilution. Collectively, the titer of antiserum was 5,210,000. Along with that, there was no signal in the negative control, indicating serum before immunization did not react with ToxA.

**Figure 4:**
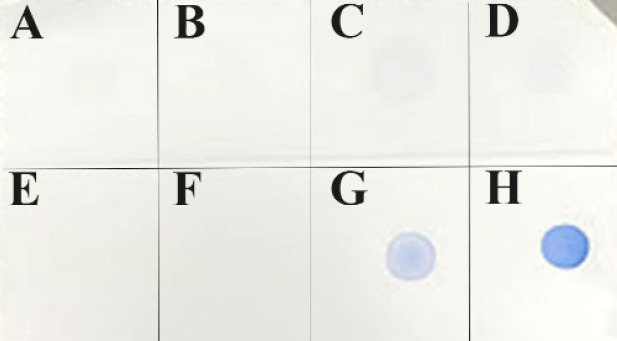
Dot blot result of specific interaction between polyclonal antibody and *V. alginolyticus* (A), *V. vulnificus *(B)*, V. cholerae *(C), *V. parahaemolyticus* XN8 (D), White Spot Syndrome virus (C)*, V. parahaemolyticus* XN89 (G), and recombinant ToxA (H).

Bacteria and virus, which usually reside in shrimp were used for evaluating the specificity of antiserum by Dot blot method (Figure 4). Result showed that only two dots of recombinant ToxA and VP_AHPND_ gave positive signals, other samples including VP_non-AHPND_ strain, *V. cholerae, V. vulnificus, V. alginolyticus*, and White Spot Syndrome virus (WSSV) showed no signal.

**Figure 5:**
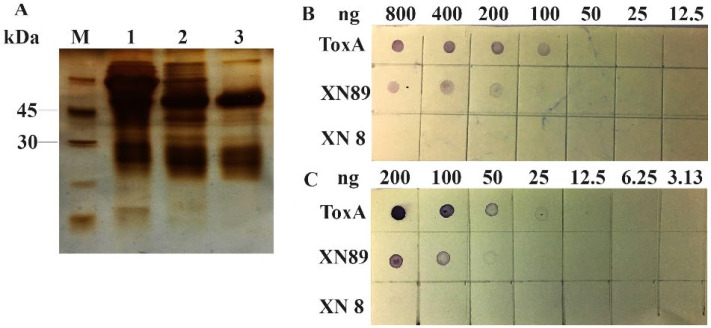
Result of antibody purification and evaluating threshold of antibody. (A) SDS-PAGE result of purification polyclonal antibody by precipitation with ammonium sulphate 60% and protein A column. M, protein ladder. 1, antisera sample. 2, precipitated proteins. 3, purified antibody. Evaluating threshold of antisera (B) and purified polyclonal antibody (C).

Antibodies in antiserum were precipitated by ammonium sulfate 50% and purified by affinity chromatography using protein A column, and analyzed by SDS-PAGE. Results showed that the precipitation fraction with 50% ammonium sulfate has not removed completely unwanted proteins (lane 2, Figure 5A). Therefore, we purified the precipitated fraction to eliminate these unwanted proteins by protein A column. The results in lane 3, figure 5A showed that, there was no band of unwanted protein as in precipitation fraction. In the presence of 1,4-Dithiothreitol (DTT), IgG was split into two clusters of protein, a heavy chain about 50kDa and light chain about 25-30kDa. Thus, polyclonal antibody was purified from antiserum, unwanted proteins were removed. 

Purified antibody was expected to give higher threshold; thus antiserum and purified antibody were again evaluated threshold using dot blot with ToxA and natural toxin. Antiserum with dilution 1/10,000 could detect 100ng ToxA and 200ng natural toxin (Figure 5B). Expectedly, purified antibody with the same concentration of antiserum reached a lower detection as it could detect 25ng ToxA and 50ng natural toxin (Figure 5C), and both antiserum and purified antibody showed no signal with VP_non-AHPND_. 

## DISCUSSION

AHPND is a highly contagious disease in shrimp with mortality rate of up to 100%. To promptly prevent, methods of detecting diseases need to be fast, accurate, and convenient. However, lateral flow kit with many advantages for *in situ* detection has not been developed yet. A body of studies shows that the disease is caused by two toxins ToxA and ToxB, thus they are both targets for disease detection. Recently, Lin et.al predicted that ToxA is necessary for receptor binding and hepatopancreatic cytotoxicity [29, 30]. Therefore, we have firstly made antibody specific for ToxA, an important component in lateral flow kit development. 

Polyclonal antibody is a potential solution because of its low production cost and ability to scale up in a short time. As antigen purity is directly linked with antibody specificity, instead of using the total protein from VP_AHPND_ as an antigen [28], we generated purified recombinant ToxA toxin. The more purity of antigen, the more specific of its antibody. Because ToxA is from bacteria, *E. coli* BL21 is the ideal host for the expression of recombinant ToxA protein. Indeed, our result showed that ToxA protein was expressed in soluble fraction at 37°C without of soluble tag [34] or decreasing culture temperature [35], which helps to produce antigen easier. High purity of antigen is necessary to improve antibody quality. Therefore, during purification by His-trap column, an amount of ToxA protein was waived in washing fraction to increase the purity up to 94.49% by only one step of purification. 

With long-term aim to develop AHPND lateral flow test kit, large amount of antibodies is needed, thus rabbit was the animal of choice instead of mouse [36, 37]. After immunization, antiserum from rabbits was evaluated. It had extremely high titer, up to 5,210,000 which has never been documented before with similar researches [38, 39] . Dot blot method was used to evaluate antigen-antibody interactions which can directly observe the result, similar to lateral flow method [28]. Bacterial strains of the *Vibrio* family, commonly reside through shrimp lifespan [40, 41], were selected as control samples for specificity evaluation [28]. Our results show that the antibody did not give any signal to control strains indicating no false-positive case. Similar result was also obtained using WSSV. We also collected natural toxin from VP_AHNPD _for evaluation, following previous method [34]. Regarding of detection threshold, current monoclonal antibody can detect 6.25ng natural toxin [28], and our polyclonal antibodies could detect up to 25ng natural toxin. It’s worth noting that in lethal test, a dose of 2ug per 1g of shrimp is capable of killing shrimp [34], thus, the threshold of detecting 25ng toxin was suitable for disease detection. 

Taken together, we have been successful in generating antibody specific to ToxA with high specificity and detection threshold. These results laid a premise for the development of AHPND detection kit based on antigen - antibody interactions.
